# Fragmented markets for older antibiotics and child formulations, Denmark, Norway, Sweden

**DOI:** 10.2471/BLT.24.292102

**Published:** 2024-12-03

**Authors:** Christine Årdal, Mohamed Gawad, Enrico Baraldi, Marianne Jahre, Charlotta Edlund

**Affiliations:** a Norwegian Institute of Public Health, P.O. Box 222 Skøyen, 0213 Oslo, Norway.; b Norwegian University of Life Sciences, Ås, Norway.; c Department of Business Studies, Uppsala University, Uppsala, Sweden.; d BI Norwegian Business School, Centre for Health Care Management, Department of Strategy and Entrepreneurship, Oslo, Norway.; e Public Health Agency of Sweden, Solna, Sweden.

## Abstract

Antibiotic resistance is one of the most urgent threats to public health. The development of antibiotic resistance can be reduced by the use of narrow-spectrum antibiotics that target specific bacteria, meaning that fewer non-harmful bacteria are killed and other harmful bacteria are not exposed to selection pressure. However, many narrow-spectrum antibiotics were introduced decades ago and therefore lack regulatory documentation in line with current standards. An additional problem for a reliable supply is that of market fragmentation, where countries with similar resistance patterns and prescribing cultures (e.g. Norway and Sweden) prioritize different formulations and strengths. For example, over half of Sweden’s highest priority paediatric antibiotics are not marketed in Denmark or Norway in the same formulations or dosages. Such market fragmentation, which can result in the annual demand of a country being smaller than batch production sizes, means that specific strengths and formulations may no longer be economical to supply. Further, once an antibiotic has been withdrawn from the market, it is difficult to attract a new supplier because of the cost of the clinical trials required to update approval of the drug. However, as resistance to antibiotics increases among populations, clinicians need access to the maximum possible range of antibiotics. Regional collaboration, that is, the harmonization of essential medicines lists (including strengths and formulations for older antibiotics) between countries, is a recommended first step towards reliable access to the necessary range of antibiotics.

## Résumé

La résistance aux antibiotiques est l’une des menaces les plus urgentes pour la santé publique. Il est possible de contrer le renforcement de la résistance aux antibiotiques par le recours à des antibiotiques à spectre étroit qui ciblent des bactéries spécifiques. Ainsi, un moins grand nombre de bactéries non nocives sont tuées et d’autres bactéries nocives ne sont pas exposées à la pression de sélection naturelle. Cependant, de nombreux antibiotiques à spectre étroit ont été introduits il y a plusieurs décennies et ne disposent donc pas d’une documentation réglementaire conforme aux normes actuelles. La fragmentation du marché constitue un problème supplémentaire pour la fiabilité de l’approvisionnement, car les pays présentant des schémas de résistance et des cultures de prescription similaires (comme la Norvège et la Suède) accordent la priorité à des formulations et à des concentrations différentes. Par exemple, plus de la moitié des antibiotiques pédiatriques prioritaires en Suède ne sont pas commercialisés au Danemark ou en Norvège dans les mêmes formulations ou dosages. Cette fragmentation du marché, qui peut avoir pour conséquence que la demande annuelle d’un pays est inférieure aux volumes de production par lots, implique que la fourniture de dosages et de formulations spécifiques peut ne plus être rentable. En outre, une fois qu’un antibiotique a été retiré du marché, il est difficile d’attirer un nouveau fournisseur en raison du coût des essais cliniques nécessaires pour actualiser l’approbation du médicament. Cependant, dans la mesure où la résistance aux antibiotiques croît au sein des populations, les cliniciens doivent avoir accès à la gamme la plus large possible d’antibiotiques. Une collaboration régionale entre les pays, c’est-à-dire l’harmonisation des listes de médicaments essentiels (y compris les dosages et les formulations d’antibiotiques plus anciens), constitue une première étape recommandée vers un accès fiable à la gamme nécessaire d’antibiotiques.

## Resumen

La resistencia a los antibióticos es una de las amenazas más urgentes para la salud pública. El desarrollo de la resistencia a los antibióticos puede reducirse mediante el uso de antibióticos de espectro reducido que se dirigen a bacterias específicas, lo que significa que se matan menos bacterias no dañinas y otras bacterias dañinas no están expuestas a la presión de selección. Sin embargo, muchos antibióticos de espectro reducido se introdujeron hace décadas y, por lo tanto, no disponen de documentación reglamentaria acorde con los estándares actuales. Otro problema para un suministro fiable es la fragmentación del mercado, donde países con patrones de resistencia y culturas de prescripción similares (por ej., Noruega y Suecia) dan prioridad a diferentes formulaciones y concentraciones. En el caso de Suecia, por ejemplo, más de la mitad de los antibióticos pediátricos de mayor prioridad no se comercializan en Dinamarca ni en Noruega en las mismas formulaciones o dosis. Esta fragmentación del mercado, que puede dar lugar a que la demanda anual de un país sea inferior a los tamaños de producción de los lotes, significa que puede dejar de ser rentable suministrar concentraciones y formulaciones específicas. Por otra parte, una vez que un antibiótico ha sido retirado del mercado, es difícil atraer a un nuevo proveedor debido al coste de los ensayos clínicos necesarios para actualizar la aprobación del medicamento. Sin embargo, a medida que aumenta la resistencia a los antibióticos entre la población, los médicos necesitan tener acceso a la mayor variedad posible de antibióticos. La colaboración regional, es decir, la armonización de las listas de medicamentos esenciales (incluidas las concentraciones y formulaciones de los antibióticos más antiguos) entre los países, es un primer paso recomendado hacia un acceso fiable a la variedad necesaria de antibióticos.

## ملخص

إن مقاومة المضادات الحيوية هي إحدى أكثر التهديدات خطورة بالنسبة للصحة العامة. ويمكن الحد من تطور مقاومة المضادات الحيوية باستخدام المضادات الحيوية ضيقة النطاق، والتي تستهدف بكتيريا معينة، مما يعني قتل عدد أقل من البكتيريا غير الضارة، وعدم تعرض البكتيريا الضارة الأخرى لضغوط الاستهداف. ومع ذلك، تم تقديم العديد من المضادات الحيوية ضيقة النطاق منذ عقود، وبالتالي تفتقر إلى التوثيق التنظيمي بما يتماشى مع المعايير القياسية الحالية. هناك مشكلة إضافية للإمداد الموثوق به وهي تجزئة السوق، حيث تقوم بعض الدول ذات أنماط المقاومة وثقافات النشرات الطبية المماثلة (مثل النرويج والسويد) بمنح الأولوية لتركيبات وقوى مختلفة. على سبيل المثال، فإن أكثر من نصف المضادات الحيوية للأطفال ذات الأولوية القصوى في السويد، لا يتم تسويقها في الدنمارك أو النرويج بنفس التركيبات أو الجرعات. مثل هذه التجزئة في السوق، والتي يمكن أن تؤدي إلى أن يكون الطلب السنوي لدولة ما أصغر من أحجام الإنتاج على دفعات، يعني أن نقاط القوة والتركيبات المحددة قد لا تكون اقتصادية للتوريد. وعلاوة على ذلك، بمجرد سحب مضاد حيوي من السوق، يصبح من الصعب اجتذاب مورد جديد بسبب تكلفة التجارب الإكلينيكية المطلوبة لتحديث الموافقة على العقار. إلا أنه مع تزايد مقاومة المضادات الحيوية بين السكان، يحتاج الأطباء إلى الوصول إلى أقصى نطاق ممكن من المضادات الحيوية. إن التعاون الإقليمي بين الدول، أي توحيد قوائم الأدوية الأساسية (بما في ذلك نقاط القوة والتركيبات للمضادات الحيوية القديمة)، يشكل خطوة أولى موصى بها نحو الوصول الموثوق إلى النطاق الضروري من المضادات الحيوية.

## 摘要

抗生素耐药性是公共卫生系统面临的最紧迫威胁之一。使用针对特定细菌的窄谱抗生素可以降低产生抗生素耐药性的几率，这意味着杀死的无害细菌更少，对其他有害细菌也不会产生选择压力。然而，许多窄谱抗生素是在几十年前开始采用的，因此缺乏符合现行标准的相关监管文件。阻碍可靠供应的另一个问题是市场分化，即具有类似耐药谱和处方文化的国家（如挪威和瑞典）的优先考虑剂型和剂量也会有所不同。例如，对于在瑞典具有最高优先级的儿科用抗生素，有一半以上未在丹麦或挪威以相同的剂型或剂量销售。这种市场分化可能导致一个国家的年需求量低于批量生产规模，这意味着可能无法以经济实惠的价格提供特定剂量和剂型的抗生素。此外，一种抗生素一旦撤出市场，就很难吸引新的供应商，因为更新药物批准文件需要承担开展临床试验的费用。然而，随着人们对抗生素的耐药性增强，临床医生需要获得尽可能多的抗生素。建议开展区域合作，即各国采用统一的基本药物清单（包括老牌抗生素的剂量和剂型），以成功迈出可确保获得各种必要抗生素的第一步。

## Резюме

Устойчивость к действию антибиотиков – одна из самых серьезных угроз для состояния здоровья населения. Развитие устойчивости к действию антибиотиков может быть снижено за счет использования антибиотиков узкого спектра действия, которые направлены на конкретные бактерии. В этом случае погибает меньшее количество полезных бактерий, а другие вредные бактерии не подвергаются давлению отбора. Однако многие антибиотики узкого спектра действия были разработаны несколько десятилетий назад и поэтому не располагают нормативной документацией, соответствующей современным стандартам. Дополнительной проблемой, препятствующей надежным поставкам, является фрагментация рынка, когда страны со схожей резистентностью и культурой назначения лекарств (например, Норвегия и Швеция) отдают предпочтение разным препаратам и их дозировкам. Например, более половины наиболее приоритетных антибиотиков для лечения детей в Швеции не продаются в Дании или Норвегии в тех же лекарственных формах и дозировках. Такая фрагментация рынка, в результате которой годовой спрос в стране может быть меньше объема серийного производства, означает, что конкретные дозировки и лекарственные формы могут оказаться нерентабельными для поставки. Кроме того, после изъятия антибиотика с рынка трудно привлечь нового поставщика из-за стоимости проведения клинических испытаний, необходимых для повторного одобрения препарата. Однако по мере роста резистентности к антибиотикам среди населения врачам необходим доступ к максимально возможному набору антибиотиков. Первым шагом на пути к надежному доступу к необходимому ассортименту антибиотиков является региональное сотрудничество, то есть гармонизация перечней основных лекарственных средств (включая дозировки и лекарственные формы старых антибиотиков) между странами.

## Introduction 

Antibiotic resistance is one of the most urgent threats to public health. The development of antibiotic resistance can be reduced by the use of narrow-spectrum antibiotics that target specific bacteria, meaning that fewer non-harmful bacteria are killed and other harmful bacteria are not exposed to selection pressure. As resistance to antibiotics increases among populations, clinicians need access to the maximum possible range of antibiotics; however, this range of access is not always available, exacerbated by the fact that some older antibiotics, like flucloxacillin, are no longer being marketed in many countries.^1^

Clinicians in the Scandinavian countries Denmark, Norway and Sweden, prescribe the highest ratio of narrow-spectrum to broad-spectrum antibiotics in Europe.^2^ However, many narrow-spectrum antibiotics were introduced decades ago, pre-dating the formation of the European Medicines Agency. Regulatory approval for these antibiotics was obtained nationally, often many decades ago, meaning that in many cases well-performed randomized clinical trials using current dosing regimens are lacking. 

The World Health Organization (WHO) classifies antibiotics as either access, watch or reserve.^3^ Access antibiotics are defined as those that “have activity against a wide range of commonly encountered susceptible pathogens while also showing lower resistance potential than antibiotics in the other groups.” Launched in the 1940s, both benzylpenicillin and phenoxymethylpenicillin are still classified as essential access antibiotics by WHO. Despite their longevity, these narrow-spectrum penicillins are still effective for common infections. For example, pharyngitis is often caused by group A *streptococci* that are susceptible to phenoxymethylpenicillin.^4^^,^^5^ However, European consumption of narrow-spectrum penicillins is decreasing as many countries move to broader-spectrum alternatives such as amoxicillin and amoxicillin/clavulanic acid,^6^ which may be necessary for other indications. 

Although certain older antibiotics are still being marketed in some countries,^1^ their levels of consumption may not support a continuous and reliable market because of product fragmentation. When the market was larger for these antibiotics, suppliers were able to provide multiple formulations, strengths and package sizes. As health-care systems shifted to newer antibiotics, often broader in spectrum, these slight variations remained, making already small markets smaller and economically tenuous. In this paper we examine the fragmentation of the antibiotic market in Scandinavia and the resulting availability, particularly paediatric formulations. We describe how the harmonization of medicines, allowing pharmaceutical companies to consolidate production to standard strengths and formulations, can facilitate improved availability.

## The Scandinavian situation

The three most commonly consumed antibiotic treatments in Norway in 2022 were phenoxymethylpenicillin, pivmecillinam and dicloxacillin, all of which are narrow-spectrum, older penicillins.^7^^,^^8^ Antibiotic surveillance reports in Denmark and Sweden do not provide consumption per substance, but report that these narrow-spectrum penicillins were the most frequently prescribed antibiotic group in 2023.^8^^,^^9^ Despite this homogeneity, these three countries (total population only around 22 million)^10^ use and prioritize slightly different antibiotics.

Public health experts in both Norway and Sweden have separately established prioritized lists of critical antibiotics (including combination therapies). In 2017, the Swedish government initiated an assessment of the antibiotics that are considered critical for public health and at risk of supply failure (based on low annual sales).^11^ The resulting list (updated in 2021) categorizes antibiotics into three groups. Category A includes those considered very valuable, for which there would be considerable clinical consequences if unavailable; category B includes those considered valuable to have access in Sweden; and category C includes those for which relatively equivalent alternatives are available.^12^

In contrast, the Norwegian government became concerned about potential medicine supply failures during the coronavirus disease 2019 (COVID-19) pandemic and acted to create a 6-month stockpile of critical medicines, including antibiotics.^13^

Both the Norwegian and Swedish lists specify critical antibiotics at the substance, formulation and strength levels, and both contain nationally withdrawn products. The two lists are relatively similar at the active substance level. The Swedish government classifies 26 substances in the A category, and the Norwegian government’s stockpile of critical medicines includes 30 substances. A total of 22 substances are included in both lists, that is, 84.6% of the Swedish list and 73.3% of the Norwegian list. However, the Swedish list includes only those medically important antibiotics that are at risk of supply failure based on low annual sales; the Norwegian stockpile includes the antibiotics benzylpenicillin, meropenem and bedaquiline, which are not considered to be at risk of supply failure by the Swedish government.^11^

This fragmentation is also apparent in the formulation and strength levels of drugs included in the priority lists. The two lists have only 13 matching antibiotic products, that is, those for which pharmaceutical companies could supply a product of identical formulation and strength. However, six of these 13 (three tuberculosis medicines, gentamicin, and oral suspension erythromycin and phenoxymethylpenicillin) are included because the Norwegian government does not stipulate strength. 

## Phenoxymethylpenicillin market

The most consumed antibiotic in Scandinavia, phenoxymethylpenicillin, has a fragmented market across the whole region. For example, 250 mg and 500 mg tablets are included in the Swedish list of priority medicines, but as of October 2024, the Norwegian list includes the 650 mg or 660 mg strengths. Indeed, the multisectoral collaboration platform Platinea found that Denmark, Finland, Iceland, Norway and Sweden favoured 10 different strengths of tablet formulations in 2023, requiring a total of 41 different package sizes.^14^ Clinicians in Denmark and Norway have traditionally prescribed tablets with strengths of 165 mg, 330 mg, 660 mg and 1 g, whereas Swedish physicians prescribe 250 mg, 500 mg, 800 mg and 1 g. 

European consumption of phenoxymethylpenicillin is declining, comprising only around one tenth of all European community penicillin consumption in 2017; much of this consumption is in the small Scandinavian markets,^6^ in which regulators have held unit prices at low levels. It is therefore unsurprising that pharmaceutical companies are withdrawing certain products from these markets. Clinicians in Denmark and Norway have recently lost access to the droplets and 165 mg tablets (for treatment of children and infants) and the 330 mg tablets. Neither country has gained access to the scored 250 mg tablet (i.e. facilitating dosages of 125 mg), meaning that there is no paediatric tablet available in Denmark or Norway. Because phenoxymethylpenicillin has a bitter taste, the lack of a paediatric tablet where taste can be masked by giving with food means that it is more difficult to administer. Without access to a paediatric tablet or the fruit-flavoured Swedish oral suspension, children in Denmark and Norway must drink the bitter oral suspension.

The dosing regimens of these older antibiotics may also differ between countries, thereby prohibiting the production of an optimal package size. For example, the antibiotic prescribing guidelines for the standard treatment of pneumonia in adults all recommend phenoxymethylpenicillin as the first-line treatment, but with different dosing regimens: 660 mg four times per day for 5 days in Denmark;^15^ 1 g four times per day for 7 days in Norway;^16^ and 1 g three times per day for 7 days in Sweden.^17^ These diverging guidelines create pressure on suppliers, requiring different strengths, package sizes and package leaflets. As a result, suppliers withdraw unprofitable first-line antibiotics such as phenoxymethylpenicillin 165 mg tablets, leaving patients (especially children) with fewer treatment options.

## Paediatric formulations

European paediatrician societies issued a position paper on medicine shortages in 2023, emphasizing that “children are a particularly vulnerable patient group with very limited pharmaceutical treatment options”, making them especially vulnerable to “adverse outcomes caused by shortages of essential medications”.^18^ This concern is exemplified in the Swedish priority antibiotic list, as almost half of the antibiotics (17/37) in category A are paediatric formulations.^12^

Since paediatric markets are generally smaller (in both sales volumes and revenues) than those for adult formulations, it is useful to understand whether countries with similar antibiotic resistance patterns^2^ utilize the same paediatric antibiotics. For this reason, we expanded our examination to include Finland and the Kingdom of the Netherlands, two countries with similar resistance patterns to Scandinavia. We assessed the national marketing authorization status through each country’s national medicines register of the 17 Swedish category A paediatric antibiotics in all five countries (Table 1). All of these antibiotics pre-date the European Medicines Agency, so none has received central marketing approval. All except two (ceftibuten and cefadroxil) are included in the European Union list of critical medicines, although this list is only at substance (not strength) level so there is no focus on paediatric formulations.^19^

**Table 1:** Comparative availability of non-tubercular child formulations classified by Swedish Public Health Agency as a category A antibiotic, 31 August 2023

Antibiotic	Spectrum		No. of marketing authorization holders				
			Denmark	Finland	Kingdom of the Netherlands	Norway	Sweden
Amoxicillin, 50 mg/mL oral suspension	Broad		2	1	5	0	2
Amoxicillin, 100 mg/mL oral suspension	Broad		0	2	2	2	4
Amoxicillin/clavulanic acid, 50 mg/mL + 13 mg/mL oral suspension	Broad		3	1	2	0	1
Azithromycin, 250 mg tablet	Broad		5	4	8	0	6
Ceftibuten, 36 mg/mL oral suspension	Broad		0	0	0	0	0
Cefadroxil, 100 mg/mL oral suspension	Broad		0	0	0	0	2
Cefixime, 20 mg/mL oral suspension	Broad		0	0	0	0	0
Ciprofloxacin, 100 mg/mL oral suspension	Broad		0	0	3	0	4
Clindamycin, 15 mg/mL oral suspension	Narrow		0	1	1	1	1
Erythromycin, 100 mg/mL oral suspension	Broad		1	0	0	2	1
Flucloxacillin, 50 mg/mL oral suspension	Narrow		0	0	0	0	1
Flucloxacillin, 125 mg tablet	Narrow		0	0	0	0	1
Metronidazole, 40 mg/mL oral suspension	Broad		1	0	1	1	1
Phenoxymethylpenicillin, 50 mg/mL oral suspension	Narrow		3	1	0	1	3
Phenoxymethylpenicillin, 250 mg tablet	Narrow		0	0	3	0	1
Sulfamethoxazole/trimethoprim, 40 mg/mL + 8 mg/mL oral suspension	Broad		0	0	1	1	2
Trimethoprim, 10 mg/mL oral suspension	Broad		1	1	0	1	1

Note: antibiotics in category A are considered very valuable, for which there would be considerable clinical consequences if unavailable.^12^

None of the 17 paediatric formulations is marketed in all five countries. Three formulations (cefadroxil and both formulations of flucloxacillin) are available only in Sweden. Two formulations (ceftibuten and cefixime) are not marketed in any of the five countries. Clinicians in Denmark, Finland and Norway each have access to seven of these 17 (41.2%), but not the same seven products. Clinicians in the Kingdom of the Netherlands, with the largest population of the five countries, has access to only nine of the 17 (52.9%). 

The high priority placed on the availability of child-appropriate antibiotics by the Swedish health ministry is demonstrable, but it may not be possible to maintain access to these priority products without multinational collaboration. The Swedish market alone is not large enough to maintain access to any unique antibiotic, as demonstrated by the market withdrawal of Category A ceftibuten in Sweden in 2017.

## Harmonization for reliable supply

The ability to use narrow-spectrum antibiotics in the Scandinavian countries is one tool that contributes to keeping antibiotic resistance levels low relative to the rest of the world.^20^^–^^22^ However, as more countries around the world adjust to the scarce supply or withdrawal of these antibiotics, their supply in Scandinavia becomes less certain. The Scandinavian countries have been pursuing separate paths to secure access to these vulnerable but essential antibiotics, particularly narrow-spectrum penicillins. The Danish government emphasizes the use of narrow-spectrum antibiotics in its national action plan on antimicrobial resistance.^23^ The Swedish government has commissioned assessments of how to maintain predictable access, including through Scandinavian collaboration.^24^^,^^25^ In contrast, the Norwegian government has considered subsidizing local production, but concluded that market-stimulating interventions in collaboration with other countries would be more effective and less costly.^26^

Although there is an undisputed need to improve the financial attractiveness of these markets (regulators in Norway and Sweden have recently increased unit prices), one of the first steps towards a predictable supply is the harmonization of local needs. Scandinavian countries have similar antibiotic resistance patterns and prescribing cultures, highlighting that the current fragmentation is not based upon patient or public health needs; instead, this fragmentation is a historical artefact of locally developed guidelines, idiosyncratic preferences and previous regulations.

Apart from the uncertainty of supply, an added pressure is that once a supplier withdraws an older antibiotic from the market, it is difficult to attract a new supplier. The documentation that would have been the basis for the original approval of the antibiotic decades ago is outdated. For example, in the health-care systems of Denmark and Norway, the indication of benzylpenicillin is “infections caused by benzylpenicillin-sensitive bacteria.” This indication would not be considered valid by today’s standards as it does not specify a site or type of infection – for example, community-acquired pneumonia. However, updating documentation in accordance with current standards is prohibitively costly, requiring expensive clinical trials; no supplier would pursue these clinical trials as investments would never be recouped with the current low-margin, low-volume products and generic competition.

## Required regional collaboration 

Relaunching formulations of older, narrow-spectrum antibiotics in countries where they are no longer available, for example, paediatric tablets of phenoxymethylpenicillin in Norway, will require at least regional collaboration, as these markets will never be large. Fortunately, collaborative efforts began in 2024 with the initiation of the second European Union joint action on antimicrobial resistance and health care-associated infections (EU-JAMRAI-2), focused on improving access to the antibiotics identified by participating countries as critical and with a vulnerable supply. In this way, like-minded countries can join efforts to improve the availability of paediatric narrow-spectrum penicillins and other discontinued antibiotics. 

Harmonization of medicines is the clear first step. If the health experts of Denmark, Norway, Sweden and other interested countries can agree on standard formulations, strengths and package sizes, suppliers could match demand with batch production sizes. Harmonization is already occurring, but not by choice; as products are withdrawn from the market, clinicians have no alternative but to prescribe the strengths and formulations that are available. This enforced harmonization means that dosing deviates not only from prescribing guidelines but also package leaflets. A much better process would be for national stakeholders to agree on the specific products (substance, formulation and strength) they want available based upon a market analysis of existing products, work together to encourage companies to market these products nationally, and update the national guidelines based upon available antibiotics. Expecting that national health systems will collaborate on common antibiotic prescribing guidelines might be unrealistic, but collaboration on harmonized supply is already occurring through EU-JAMRAI-2. The health experts of all five countries in Table 1 (as well as those in Belgium, Bulgaria, France, Iceland and Slovenia) have identified phenoxymethylpenicillin as a focus product, beginning the process of harmonization.^27^
